# Experimental protothecosis in a murine model

**DOI:** 10.1371/journal.pntd.0014312

**Published:** 2026-05-11

**Authors:** Angelika Proskurnicka, Karolina Duk, Tomasz Hutsch, Grażyna Hoser, Robert Wrzesień, Tomasz Skirecki, Jacek Bielecki, Tomasz Jagielski

**Affiliations:** 1 Department of Medical Microbiology, Faculty of Biology, Institute of Microbiology, University of Warsaw, Warsaw, Poland; 2 Veterinary Diagnostic Laboratory ALAB Bioscience, Warsaw, Poland; 3 Department of Pathology and Veterinary Diagnostics, Institute of Veterinary Medicine, Warsaw University of Life Sciences, Warsaw, Poland; 4 Department of Translational Immunology and Experimental Intensive Care, Centre of Postgraduate Medical Education, Warsaw, Poland; 5 Central Laboratory of Experimental Animals, Medical University of Warsaw, Warsaw, Poland; Albert Einstein College of Medicine, UNITED STATES OF AMERICA

## Abstract

*Prototheca* spp. are unicellular, yeast-like microalgae, and the only plant lineage known to cause opportunistic infections in vertebrates, including humans. The aim of the study was to establish a comprehensive murine model of protothecosis to systematically investigate the influence of *Prototheca* species, inoculum dose, infection route, and host immune status on disease development and severity. Three pathogenic (*P. wickerhamii*, *P. bovis*, *P. ciferrii*) and one saprophytic (*P. stagnora*) species were used to infect immunocompetent or athymic mice. A total of 324 animals were split into 54 groups according to inoculum size (10⁶ or 10⁷) and infection route (subcutaneous, intramammary, or intraperitoneal). Six weeks post-infection, mice were euthanized, and organs were collected for microbiological and histopathological analyses. All four *Prototheca* species produced infection in mice, yet the infection potential differed considerably between the species. *P. ciferrii* exhibited the highest infection rate (61.1%), followed by *P. bovis* (45.8%) and *P. wickerhamii* (31.9%), whereas *P. stagnora* was the least virulent (11.1%). Athymic mice were markedly more susceptible compared to wt mice (45.1% *vs*. 29.9%) and more prone to develop multifocal infections. Higher inocula (10⁷) increased infection yield, while the inoculation route influenced the infection site but not its severity. In the cytokine profile, IL-10 and TNF-α were most prominently elevated, with significantly higher levels in wt than in athymic mice. This study highlights three hallmarks of protothecal disease: a species-specific infection pattern, chronic, asymptomatic infection as the clinical manifestation, and the essential role of host immunity in determining disease trajectory and severity.

## Author summary

*Prototheca* spp. are colorless microalgae and the only plants known to cause infections in animals and humans. However, there is an important gap in knowledge on the biology of these algae, including their pathogenicity and interactions with the host. In this study, a comprehensive murine model of protothecosis was developed to investigate how different *Prototheca* species, infectious dose, route of exposure, and host immune status influence disease development. Our findings demonstrate that *Prototheca* algae exhibit distinct infection patterns, with the highest infection rates observed for *P. ciferrii* and the lowest for *P. stagnora*, a species previously considered exclusively saprophytic. Higher infectious doses and an immunodeficient host phenotype were associated with increased infection rates and a greater propensity for dissemination. Analysis of the immune response showed that IL-10 and TNF-α were the most prominently elevated cytokines, suggesting their potential role in the control of infection. In addition, the combination of microbiological and histopathological approaches improved the detection of *Prototheca* in host tissues compared with either method alone. Overall, this study provides a robust experimental framework for investigating protothecosis and highlights the importance of both pathogen- and host-related factors in shaping infection outcomes.

## Introduction

*Prototheca* species are unicellular, achlorophyllous, yeast-like microalgae thriving in a wide range of environmental habitats, typically with high moisture and organic matter content [1]. These organisms normally live a saprophytic lifestyle, but can become opportunistic pathogens and cause a variety of pathologies in both animals and humans, collectively referred to as protothecosis [1]. There was a century-long debate over the taxonomic status of *Prototheca* until it reached a final consensus with their placement in the Trebouxiophyceae class within the Chlorophyta lineage. *Prototheca* have been accepted to represent descendants of green algae of the genus *Chlorella*, which, during their evolutionary history, have lost the ability to photosynthesise and changed to a heterotrophic metabolism [1,2]. The current taxonomic classification of *Prototheca* has been built on the mitochondrially-encoded *cytb* gene, with a total of 18 distinct species described [3,4]. Five of these species have been implicated in animal protothecosis (*P. blaschkeae*, *P. bovis*, *P. ciferrii*, *P. cutis*, *P. wickerhamii*) [5–7], whereas the same five species and *P. miyajii* have been involved in human infections [8–10].

The most prevalent form of animal protothecosis is bovine mastitis, usually recognized as a chronic, subclinical infection, for which culling is the only control strategy, since no effective treatments exist. The disease has a global distribution and incurs heavy economic losses to the dairy industry and animal welfare [7,11–13]. Contrastingly, human protothecosis is a rare yet an emerging infection, whose incidence has been on the rise over the last few decades [8,14]. Typically, it manifests under three major clinical forms, namely cutaneous, articular (olecranon bursitis), and disseminated (or systemic) infections. Patients suffering from different types of immunosuppression are most commonly affected [8,10]. Of particular concern are systemic infections, for which the mortality rate is exceptionally high, exceeding 50% on average [14]. A hallmark feature of *Prototheca* infections is their refractoriness to most of the therapeutic regimens currently available with virtually no mechanisms behind this resistance disclosed [8,10,14,15].

Although *Prototheca* were first recognized as pathogens in the early 1950s [16], rarely have they been studied scientifically. Therefore, there is a huge knowledge gap on *Prototheca* pathobiology and virulence mechanisms. To the best of authors’ assessment, only slightly more than 20 studies have addressed the problem of *Prototheca* pathogenicity with the use of animal models [17–40]. In five of these studies, animal challenge with the algal culture was part of the diagnostic path described in case reports [35–39]. All *in vivo* studies have been reviewed and summarized in Table 1. Among several animal species experimentally inoculated with *Prototheca* algae were rabbits [19,22,37], guinea pigs [19,22,23,35,37], rats [22,37,40], rhesus monkey [40], mice [17,18,20–23,25–30,32–34,36–40], and dairy cows [20,24,31]. Recently, a murine model for studying *Prototheca* bovine mastitis has been proposed [32]. The inoculum dose required to cause an infection, differed across studies, ranging from 40 to 4.5 × 10⁸ cells [20,24]. The most frequently exploited route of inoculation was the intraperitoneal route (I.P.) [19–23,25,30,32,36,37,39], followed by subcutaneous (S.C.) [19,20,22,26,28,29,32,37], intramammary (I.M.) [17,18,20,24,31,32,34], intratesticular (I.T.) [19,22,25,38], intravenous (I.V.) [19,22,25,27], and intradermal (I.D.) [23,28,29,35] exposure. Of these, I.M. and I.T. routes proved most efficacious, invariably leading to an infection. The I.D., I.P., I.V., S.C. infection routes were somewhat less effective, but still resulted in infection in at least half of the animals challenged [19–21,25–30,32,36,39]. In single cases, alternative inoculation methods were employed, such as intramuscular (I.MS.) [20], intratracheal (I.TR.) [21], oral (P.O.) [40], and also topical (T.) [33], transdermal (T.D.) [32], and intraocular (I.O.) [37] delivery, each but the latter two producing an infection.

**Table 1:** Studies using animals in experimental protothecosis – literature review.

No.	Year^1^	Host	Host strain/ breed^2^	Inno-culation site^3^	Infection dose (CFU)^4^	*Prototheca* species^5^	Observation time^6^	Infection^7^	Infected organs^8^	Investigation method^9^			Treatment^10^	Ref.
**H.-P.**	**CUL.**	**MOL.**
1.	1941	Rabbits	UNS.	I.V.	UNS.	*P. ciferrii* ^P^	1 m	+^L^	Lungs, heart	+	–	–	–	[19]
				I.T.			1.5 m	+^L^	Testis	+	–	–		
		Guinea pigs		I.T.			10 d	+^L^	Testis	+	–	–		
				I.P.			5 d	+^L^	UNS.	+	+	–		
				S.C.			>2 m	+	–	–	–	–		
				I.T.			17 d	+^L^	Testis	+	–	–		
2.	1964	Guinea pigs	UNS.	I.D.	UNS.	*P. zopfii* ^H^	UNS.	–	–	UNS.	UNS.	UNS.	–	[35]
3.	1966	Mice	UNS.	I.P.	UNS.	*P. wickerhamii* ^H^	UNS.	+	Spleen, liver	–	+	–	–	[36]
4.	1968	Mice	UNS.	I.P. S.C. I.O. ^R^	UNS.	*Prototheca* sp. ^H^	UNS.	–	–	UNS.	UNS.	UNS.	–	[37]
		Rats	UNS.					–	–					
		Rabbits	UNS.					–	–					
		Guinea pigs	UNS.					–	–					
5.	1968	Cows	UNS.	I.M.	3.45 × 10^8^, 4.5 × 10^8^	*P. zopfii* ^A^	1-12 d	+^2/3^	Milk samples	–	+	–	+ (OTC)	[20]
					5 × 10^7^, 4 × 10^7^	*P. moriformis* ^B^		+						
		Mice	Albino	S.C. I.MS. I.P.	1-2 × 10^8^	*P. zopfii* ^A^	2-50 d	+ (S.C.; I.P.)	UNS.	+	–	–	–	
						*P. moriformis* ^B^		+ (S.C.; I.MS.)	UNS.	+	–	–	–	
6.	1971	Mice	UNS.	I.P.	1 × 10^3^, 1 × 10^4^	*Prototheca* sp.^C^	UNS.	–	–	–	–	–	–	[21]
						*Prototheca* sp.^A^		+	Kidneys	+	–	–	–	
				I.TR.	1 × 10^5^	*Prototheca* sp.^A^		+^L^	Lungs	+	–	–	–	
7.	1980	Mice	UNS.	I.T. I.P. I.V. S.C.	5 McF suspension: 0.1 mL I.T.; 1 mL I.P.; 0.5 mL I.V.; 0.1 mL. S.C.	*P. zopfii* *P. wickerhamii* *P. stagnora*	15-60 d	+ (I.T.)	Testis	+	+	–	–	[22]
		Rats	UNS.											
		Rabbits	UNS.		5 McF suspension: 0.5 mL I.T.; 2 mL I.P.; 1 mL I.V.; 0.1 mL. S.C.									
		Guinea pigs	UNS.											
8.	1981	Guinea pigs	UNS. ^WT, N^	I.D. I.P.	UNS.	*P. wickerhamii* ^H^	UNS.	–	UNS.	UNS.	UNS.	–	–	[23]
		Mice	UNS. ^A^					–						
9.	1983	Mice	UNS.	P.O.	UNS.	*P. zopfii* *P. wickerhamii*	UNS.	-^*^	Faeces	–	–	–	–	[40]
								-^L**, *^						
		Rats	UNS.				UNS.	-^*^						
		Rhesus monkey	UNS.				UNS.	-^*^						
10.	1984	Cows	UNS.	I.M.	4 × 10 - 4.8 × 10^2^	*P. zopfii* ^B^	21 d	+	Mammary glands, lymph nodes, milk samples	+	+	–	–	[24]
11.	1985	Mice	UNS.	I.P. I.V. I.T.	5 McF suspension: 1 mL I.P.; 0.5 mL I.V; 1 mL I.T.	*P. zopfii* ^B^	15 d	+	Testis	+	–	–	–	[25]
			UNS. ^I^						Testis, liver					
12.	1991	Mice	ICR albino	S.C.	2.5-4 × 10^6 2-6M^	*P. wickerhamii* ^H^	2-6 m	+^3/8^	Skin	+	+	–	–	[26]
			Balb/c					+						
13.	1994	Mice	Balb/c ^I^	I.V.	1 × 10^3^	*P. zopfii* ^B^	7 d	-^1/5^	Kidneys, heart, liver	+	+	–	–	[27]
					1 × 10^4^			+^4/5^	Kidneys, brain, heart, liver					
					1 × 10^5^			+	Kidneys, brain, heart, liver, lungs					
					1 × 10^6^			+						
					1 × 10^7^			+^L^						
14.	1995	Mice	ICR albino	S.C. I.D.	2.5-4 × 10^6 6M^	*P. wickerhamii* ^H^	1 w - 6 m	+^3/6^	Skin	+	–	–	–	[28]
			Balb/c					+						
15.	1995	Mice	ICR albino	S.C. I.D.	2.5-4 × 10^6 3-4M^	*P. wickerhamii* ^H^	3-4 m	+	Skin	+	–	–	–	[29]
			Balb/c											
16.	2000	Mice	Kuming	I.T.	3 × 10^4^	*P. zopfii* ^H^	3 w	+	Testis	–	+	–	–	[38]
17.	2009	Mice	Balb/c	I.P.	1 × 10^6^	*P. wickerhamii* ^T^	10, 15, 20 d	+^6/12^	Lymph nodes, skin, liver, pancreatis, diaphragm, intestines	+	+	–	–	[30]
						*P. zopfii* ^B^		+^10/12^						
18.	2010	Mice	CD-1	I.P.	1 × 10^6^	*P. bovis* ^H, Z2^	4 w	+	Lymph nodes	+	–	–	–	[39]
19.	2011	Cows	Holstein	I.M.	5 × 10^7^	*P. ciferrii* ^B^	10 d	+	Mammary glands	+	–	–	–	[31]
20.	2013	Mice	Balb/c ^P^	I.P. S.C. T.D. I.M.	1 × 10^3^, 1 × 10^4^, 1 × 10^5^, 1 × 10^6^, 1 × 10^7^	*P. bovis* ^B, Z2^	1, 3, 5, 7, 9 d	+ (I.P.; I.M.)	Abdominal nodules (I.P.); mammary glands (I.M.)	+	+	–	+ (GEN)^I^	[32]
21.	2014	Mice	Balb/c ^I^	T.	5 × 10^5 3D^	*P. bovis* ^B, Z2^	21 d	+	Skin	+	+	–	+ (*Mentha piperita*; *Saturenja hortensis* essential oils; NYT, AMB) ^I^	[33]
22.	2020	Mice	C57BL/6 ^L^	I.M.	1 × 10^4^	*P. bovis* ^B^	4 d	+	Mammary glands	+	–	–	–	[18]
23.	2020	Mice	C57BL/6 ^L^	I.M.	1 × 10^4^	*P. bovis* ^B, Z2^	4 d	+	Mammary glands	+	+	+	–	[17]
24.	2022	Mice	C57BL/6 ^P^	I.M.	4 × 10^4^	*P. bovis* ^B^	12, 24 h	+	Mammary glands	+	–	–	+ (NAC) ^I^	[34]

^**1**^ Year of publication of the report;

^**2**^ Specific features of infected animals (if provided): ^A^ = athymic; ^I^ = immunosuppressed by injection of dexamethasone or prednisolone; ^L^ = lactating; ^N^ = neutropenic; ^P^ = pregnant; ^WT^ = wild type; UNS. = unspecified;

^**3**^ Inoculation route: I.D. = intradermal; I.M. = intramammary; I.MS. = intramuscular; I.O.^R^ = intraocular (only rabbits); I.P. = intraperitoneal; I.T. = intratesticular; I.TR. = intratracheal; I.V. = intravenously; P.O. = *per os* (oral administration of food contaminated with *Prototheca* sp.); S.C. = subcutaneous; T. = topical administration; T.D. = transdermal (damaged skin infection);

^**4**^ Infection dose was calculated, if possible, and presented as a total number of algal cells (CFU); the superscripts indicate days (^XD^) and months (^XM^), over which injection was performed daily and weekly, respectively; UNS. = unspecified;

^**5**^ Z2 = *P. zopfii* gen. 2 (currently *P. bovis*), according to the original identification; Origin of strain (if provided): ^A^ = animal origin (other than bovine mastitis); ^B^ = bovine clinical mastitis; ^C^ = clinical sample (exact origin was not specified); ^H^ = human patient; ^P^ = potato; ^T^ = type strain; if no index is provided for a given strain, its exact origin was not specified in the study;

^**6**^ Post infection observation time: d = days; h = hours; m = months; w = weeks; y = years; UNS. = unspecified;

^**7**^ Infection development: + = infection post inoculation; – = no infection; ^L^ = lethal effect of infection (animal died before the end of the experiment); numbers in the superscript define the fraction of animals that developed infection; *Transient presence of *Prototheca* sp. in feces was observed; no persistent intestinal colonization occurred; **Lethal intestinal obstruction observed in animals fed with high *Prototheca* doses (UNS.);

^**8**^ Infected organs, organs in which the presence of *Prototheca* algae was confirmed; in some articles, only one organ (tissue) was investigated;

^**9**^ Identification method: H.-P. = histopathology; CUL. = culture; MOL. = molecular; UNS. = unspecified;

^**10**^ Treatment: + = treatment with some compound was tested; – = no treatment was tested; ^I^ = improvement (when some of the symptoms of the infection disappeared in the treated group of animals); AMB, amphotericin B; GEN, gentamycin; NAC, N-acetyl-L-cysteine, NYT, nystatin; OTC, oxytetracycline.

Of all *in vivo* studies thus far performed, only four have investigated the pathogenic potential of *Prototheca* algae in a comparative manner, that is using two or more *Prototheca* species [20,22,30,40]. One such study showed that *P. zopfii* (currently *P. bovis* or *P. ciferrii*) was twice as successful in causing infections compared to *P. wickerhamii* [30]. All but four *in vivo* studies employed immunocompetent animals. In one such investigation, neutropenic guinea pigs and athymic mice were used [23], whereas the remaining three involved mice that were compromised by exposure to either prednisolone [25,27] or dexamethasone [33].

In rare instances where replicable animal models, infection doses, and routes were used, the results varied considerably between the studies [20,22,27,32,37]. The discrepancies in the findings on the pathogenic capacity of *Prototheca* spp. largely arise from the variability in experimental approaches. Earlier *in vivo* studies differed significantly in their experimental parameters, including the algal strain, infection dose, infection route, host species, and the spectrum of tissues examined. This methodological variability has led to inconsistent and often irreproducible results, making meaningful comparisons across studies challenging or even unfeasible. Moreover, the very narrow scale of the previous investigations has further hampered gaining a coherent view of the *Prototheca* infectivity potential.

The only consistent pattern that emerged throughout most of the studies was that the frequency of infection and host mortality conspicuously increased with higher inoculum loads [21,27,32]. Still, the limitations in the design of previous studies preclude determination of the impact of other experimental parameters, including the algal species and the host species on the infection development and severity.

The present study was conceived to address these limitations through launching, for the first time, a large-scale, multivariable investigation, involving different types of pathogens, host species, inocula, and routes of infection. The purpose of the study was to elucidate the role of these essential experimental components on the induction and extent of *Prototheca* infection in the *in vivo* model.

## Materials and methods

### Ethics statement

This study was approved by the Local Ethical Committee for Experiments on Animals at the Warsaw University of Life Sciences, Warsaw, Poland (Approval no. WAW2/014/2021 of January 27^th^, 2021).

### Prototheca strains

Four typical *Prototheca* sp. strains: three of the clinical origin (*P. bovis* SAG 2021, *P. ciferrii* SAG 2063, *P. wickerhamii* ATCC 16529) and one environmental strain (*P. stagnora* ATCC 16528) were used. The strains were cryopreserved using Viabank Bacterial Storage Beads (MWE Medical Wire, United Kingdom) at -80°C. To revive the strains, a loopful (10 μL) of the frozen culture was streaked onto Sabouraud Dextrose Agar (SDA) (Biomaxima, Poland) plates, which subsequently were incubated at 30°C for 72 hours under aerobic conditions. To prepare the inoculation doses, a loopful of colonies was dissolved in sterile Phosphate Buffered Saline (PBS; pH = 7.4). The number of *Prototheca* sp. cells in the solution was then calculated using an improved Neubauer counting chamber. The prepared solution was adjusted by PBS to achieve the algal concentration of 5 × 10^6^ or 5 × 10^7^ CFU/mL.

### Mice

The study sample included a total of 324 female, 8-week-old mice, split into two cohorts of equal size (162 subjects), each representing a different genotype. The first cohort included wild-type Balb/cAnNRj mice, referred to as wild-type (wt), whereas the second cohort consisted of immunodeficient mice (Balb/cAnN-Foxn1nu/nu/Rj) referred to as nude or athymic mice.

All mice were purchased from Janvier Labs (France), delivered to and housed in the Central Laboratory of Experimental Animals of the Medical University of Warsaw. Once arrived, the animals were quarantined for at least 1 week before use to adapt to the new housing conditions. The mice were kept in groups of six animals (i.e., each experimental group separately) in transparent Sealsafe Plus GM500 IVC cages (Tecniplast, Italy) providing a minimum area of 0.05 m^2^. Rooms where cages were placed were equipped with mechanical ventilation (15 changes per hour), regulated daylight (12 hours day/12 hours night), temperature ranging from 20°C ± 4°C, and humidity maintained at 55 ± 10%. Food (Altromin 1318 feed (Animalab, Poland)) and water were available *ad libitum*. Cages, feed, water, and enrichment materials were sterilized by autoclaving.

**Fig 1:**
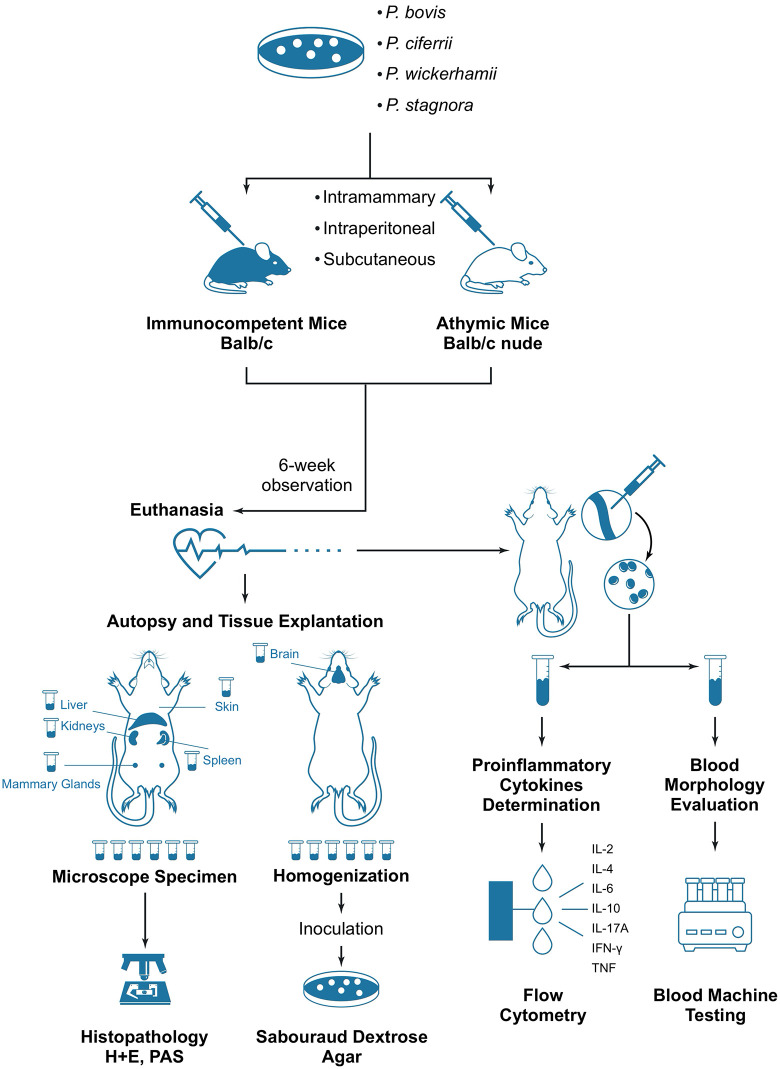
Experimental path in the murine model of *Prototheca* infection conceived in this study.

Mice of each strain were split evenly into 27 groups of 6 subjects per group (54 groups in total), depending on the inoculum (algal strain or PBS as a control), the challenging dose (a 0.2-mL aliquot of the algae containing 5 × 10^6^ or 5 × 10^7^ CFU/mL, or an equal volume of PBS), and the inoculation route (subcutaneous, intramammary, and intraperitoneal). The inocula were injected using one-milliliter syringes with a replaceable needle to either subcutaneous tissue in the neck area, the 4^th^ left mammary gland, or intraperitoneally in the lower abdominal area. A schematic diagram of the experimental path is presented in Fig 1.

### Examination and sampling

Over a 6-week experimental period, the animals were observed for behavioral and anatomical changes. Their weights were recorded twice, i.e., on the day of inoculation and on the terminal day. Humane endpoints and criteria for earlier euthanasia were defined solely as precautionary measures and were not reached during the experiment. This included body weight loss exceeding 20%, markedly reduced activity, significantly decreased food intake, injection-related complications causing pain or distress, or other severe adverse signs such as bleeding, pallor of the skin or mucous membranes, or respiratory difficulties.

**Fig 2:**
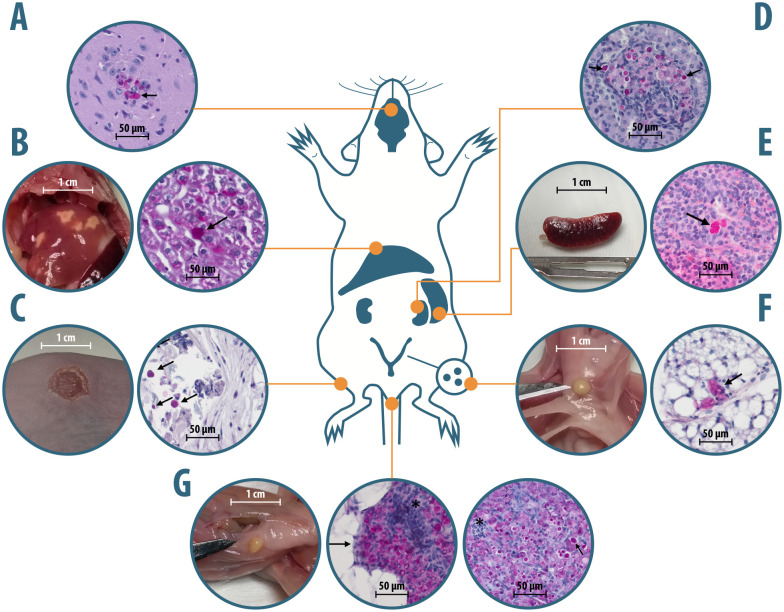
Macroscopic and histopathology-based microscopic changes upon *Prototheca* sp. infection in the brain (A), liver (B), skin (C), kidneys (D), spleen (E), mammary glands (F), and abscesses at the injection site (G).

Six weeks post-inoculation, mice were first anaesthetized by inhalation of isoflurane and euthanized by dislocation of the cervical vertebrae. Immediately after euthanasia blood was collected directly from the heart using one-milliliter disposable syringes, and transferred to the test tube containing EDTA as an anticoagulant. Organs were explanted using sterile instruments in the following order: brain, liver (2 lobes), kidneys, spleen, skin (2 cm^2^), and mammary glands. If applicable, additional lesions (e.g., abscesses) were also removed. The collected organs were weighed and divided into two portions; one was fixed in 10% formalin, while the other immersed in PBS (pH = 7.4) solution, and sent for histopathological and microbiological examination, respectively. Every sample collected was immediately placed on ice until further analysis. During post-mortem examination, macroscopic changes were systematically assessed, with particular focus on the six major organs analyzed in the study (Fig 2).

### Histopathology

The formalin-fixed tissues were rinsed in water, then dehydrated through a series of graded ethanol and xylene solutions, and finally embedded in paraffin for histopathological analysis. Once embedded in paraffin blocks, the tissue samples were sectioned using a microtome and stained with the Hematoxylin-Eosin (HE) and Periodic-Acid-Shiff (PAS) methods with a commercial kit (04–130802, Bio-Optica, Italy). Histopathological evaluations were performed by three veterinary pathologists (ALAB bioscience, Poland) using an Axiolab A5 microscope (Zeiss, Germany), and following diagnostic criteria and guidelines offered by the International Harmonization of Nomenclature and Diagnostic Criteria (INHAND; S1 Table). Based on INHAND criteria, a scalar assessment of the intensity of the identified histopathological changes was performed. Particular attention was paid to the presence of morula or thick-walled structures in both HE and PAS staining or the presence of inflammatory processes (Fig 2). Gradings of inflammation-related pathological changes were converted into the Relative Inflammatory Severity Index (RISI), defined as the number of points assigned divided by the maximum possible score.

### Microbiological examination

The tissue samples in PBS were subjected to microbiological analysis. For this purpose, 4-5 glass beads were added to each tube, and the samples were homogenized in the TissueLyser II (Qiagen, Germany) 3 times for 30 seconds at maximum power (shaking frequency, 30 Hz). Only skin explants were homogenized manually with the Omni TH device (Omni International, USA) until the suspension was homogeneous. The obtained homogenates were serially diluted and plated onto SDA plates. Colony Forming Units (CFU) were calculated after 72 hours of incubation at 30°C, normalized to tissue weight, and expressed as CFU/g. These values were determined solely for the organs. Inflammatory lesions (e.g., abscesses) were evaluated qualitatively (presence/absence of algae), as accurate weighing was not feasible due to excision with a wide margin of healthy tissue to avoid its rupture.

### Interpretation and comparison of diagnostic results

**Fig 3:**
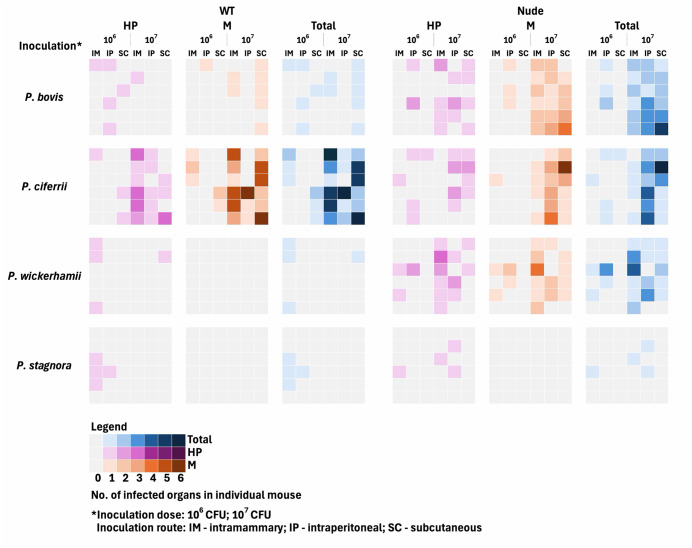
Distribution of organs affected in individual mice, of either wt or nude phenotype, upon *Prototheca* sp. infection.

*Prototheca* infection was considered to be induced in a mouse when algae were detected either on microbiology or histopathology evaluation. Infection rate (IR) was defined as the proportion of inoculated animals that developed *Prototheca* infection. Apart from six internal organs that were routinely examined (i.e., brain, kidneys, liver, mammary glands, skin, and spleen), purulent lesions (typically abscesses at the injection site) were also recorded upon necropsy. These lesions were tested only microbiologically, as they could not be divided. Thus, they were excluded from the analysis comparing the diagnostic performance of histopathology and microbiological examination. Sensitivity of each method was calculated as the proportion of true-positive cases in a given method out of all positives in both methods. A graphical comparison of detection effectiveness and the corresponding IRs are presented in Fig 3.

### Blood morphology and cytokine profiling

Blood samples collected into EDTA tubes were used for hematological analysis performed by an automated cell counts analyzer Sysmex XN-1500 (Sysmex Polska, Poland) and cytological smears stained by the May-Grünwald-Giemsa method. The following parameters were measured: red blood cells (RBC) [T/L], hemoglobin (HGB) [g/dL], hematocrit (HCT) [%], mean corpuscular volume (MCV) [fL], mean corpuscular hemoglobin (MCH) [pg], mean corpuscular hemoglobin concentration (MCHC) [g/dL], blood plates (PLT) [g/L], red cell distribution width – coefficient of variation (RDW-CV) [%], erythroblasts (EB) [%; g/L], white blood cells (WBC) [g/L], neutrophils (NEU) [%; g/L], lymphocytes (LYM) [%; g/L], monocytes (MONO) [%; g/L], eosinophils (EOS) [%; g/L], basophils (BAS) [%]. Cytological evaluation of blood was performed manually by making smears using the wedge technique and staining using. Microscopic studies were performed using an Axiolab A5 microscope and associated camera, and the ZEN 3.0 blue edition program (Zeiss, Germany).

Following hematological analysis, the residual samples were centrifuged to obtain plasma fractions, which were stored at -80°C until further analysis. Subsequently, plasma fractions were analyzed by flow cytometry (FC), to measure concentration of seven cytokines, including 5 interleukins (IL-2, IL-4, IL-6, IL-10, IL-17A), interferon-gamma (IFN-γ), and tumor necrosis factor (TNF). All assays were carried out in duplicate according to the manufacturer’s protocols using the Cytometric Bead Array (CBA) Mouse Th1/Th2/Th17 CBA Kit (BD Biosciences, USA). The data obtained were further analyzed with FCAP Array software recommended by the manufacturer (BD Biosciences, USA). Cytokine levels measured in uninfected control mice provided a reference for interpreting infection-induced immune responses.

### Statistical analysis

Statistical analyses were performed in R version 4.4.0 (R Core Team 2024). For all variables in dataset descriptive statistics were calculated. Outliers in the dataset were detected by box-plot method and Rosner’s Test for Outliers and excluded for further analyses.

For categorical variables Pearson’s Chi-squared test with Yates’ continuity correction, Fisher’s Exact test for count data were performed, depending on fulfilling test assumptions. For quantitative variables linear correlation was calculated by Pearson correlation coefficient, or depending on fulfilling test assumptions, ANOVA, U-Mann Whitney or Kruskal-Wallis test were performed. Before performing tests of group comparisons, assumptions of normality, homogeneity of variances and lack of autocorrelation were checked. Additionally, Cohen’s Kappa coefficient was calculated to assess the agreement between the results obtained by microbiological and histopathological methods. Depending on the results, a proper group comparison test was performed. A significance level of *p* < 0.05 (* *p* < 0.05; ** *p* < 0.01; *** *p* < 0.001; **** *p* < 0.0001) was considered significant for all analyses.

## Results

### Experimental infection

**Fig 4:**
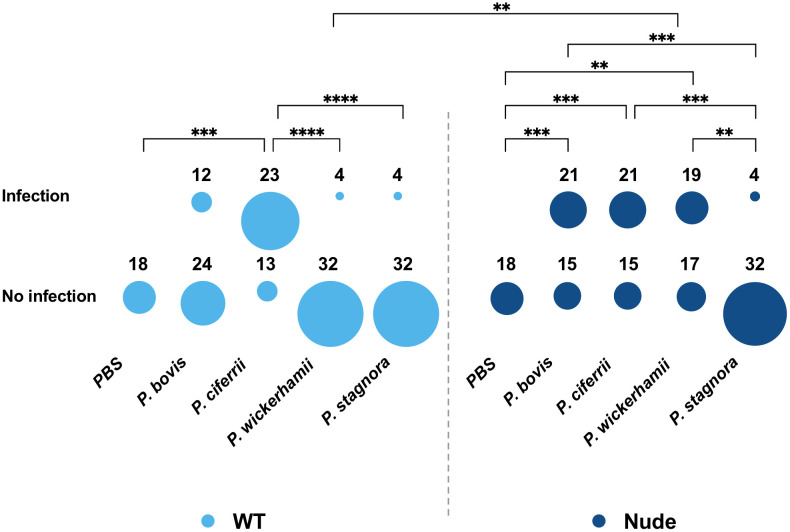
Number of mice with and without developed infection following inoculation with different *Prototheca* species, categorized by mouse phenotype (wt or nude).

*Prototheca* experimental infection could be established in nearly a third (29.9%; 43/144) of wt mice and almost a half (45.1%; 65/144) of nude mice (*p* < 0.01). These animals showed the presence of algae in at least one of the tissues examined, as evidenced upon histopathology or microbiological examination (Fig 3 and Table 2). The infection efficiency varied significantly, depending on the *Prototheca* species used for inoculation (*p* < 0.001). Overall, *P. ciferrii* accounted for the highest infection rate (44/72; 61.1%), followed by *P. bovis* (33/72; 45.8%), and *P. wickerhamii* (23/72; 31.9%). Only a small proportion (8/72; 11.1%) of inoculations with *P. stagnora* produced infection. This species ranking was the same for the two (wt and nude) mouse cohorts analyzed separately. Marked differences were observed between *Prototheca* pathogenic species (*P. ciferrii*, *P. bovis* and *P. wickerhamii*) whenever wt mice were challenged (IR of 63.9 *vs*. 33.3 *vs*. 11.1%, respectively; *p* < 0.001). This was not reproduced among nude mice, for which the IRs with all the three species were very similar (IR of 58.3% for *P. ciferrii* and *P. bovis,* and 52.8% for *P. wickerhamii*; Fig 4 and Table 2).

**Table 2:** Distribution of infection in wt and nude mice according to *Prototheca* species and route of inoculation.

Mouse strain	wt^1^												
**Species**	***P. bovis***			***P. ciferrii***			***P. wickerhamii***			***P. stagnora***			**Total** ^**3**^
**Route^2^**	**I.M.**	**I.P.**	**S.C.**	**I.M.**	**I.P.**	**S.C.**	**I.M.**	**I.P.**	**S.C.**	**I.M.**	**I.P.**	**S.C.**	
Abscess	0 (0%)	0 (0%)	5 (41.7%)	0 (0%)	4 (33.3%)	8 (66.7%)	0 (0%)	0 (0%)	0 (0%)	0 (0%)	0 (0%)	0 (0%)	17 (11.8%)
Brain	0 (0%)	1 (8.3%)	0 (0%)	2 (16.7%)	0 (0%)	0 (0%)	0 (0%)	0 (0%)	0 (0%)	0 (0%)	0 (0%)	0 (0%)	3 (2.1%)
Kidney	0 (0%)	0 (0%)	0 (0%)	3 (25%)	1 (8.3%)	3 (25%)	0 (0%)	0 (0%)	0 (0%)	0 (0%)	0 (0%)	0 (0%)	7 (4.9%)
Liver	0 (0%)	0 (0%)	0 (0%)	4 (33.3%)	1 (8.3%)	2 (16.7%)	0 (0%)	0 (0%)	0 (0%)	0 (0%)	0 (0%)	0 (0%)	7 (4.9%)
Mammary gland	3 (25%)	2 (16.7%)	0 (0%)	7 (58.3%)	3 (25%)	3 (25%)	3 (25%)	0 (0%)	1 (8.3%)	0 (0%)	1 (8.3%)	0 (0%)	23 (16%)
Skin	0 (0%)	0 (0%)	1 (8.3%)	5 (41.7%)	1 (8.3%)	8 (66.7%)	0 (0%)	0 (0%)	0 (0%)	3 (25%)	0 (0%)	0 (0%)	18 (12.5%)
Spleen	0 (0%)	0 (0%)	0 (0%)	5 (41.7%)	1 (8.3%)	2 (16.7%)	0 (0%)	0 (0%	0 (0%)	0 (0%)	0 (0%)	0 (0%)	8 (5.6%)
**No infection**	**9 (75%)**	**9 (75%)**	**6 (50%)**	**3 (25%)**	**7 (58.3%)**	**3 (25%)**	**9 (75%)**	**12 (100%)**	**11 (91.7%)**	**9 (75%)**	**11 (91.7%)**	**12 (100%)**	**101 (70.1%)**
**Infection (total)**	**3 (25%)**	**3 (25%)**	**6 (50%)**	**9 (75%)**	**5 (41.7%)**	**9 (75%)**	**3 (25%)**	**0 (0%)**	**1 (8.3%)**	**3 (25%)**	**1 (8.3%)**	**0 (0%)**	**43 (29.1%)**
**Single organ infection**	**3 (25%)**	**3 (25%)**	**6 (50%)**	**3 (25%)**	**3 (25%)**	**2 (16.7%)**	**3 (25%)**	**0 (0%)**	**1 (8.3%)**	**3 (25%)**	**1 (8.3%)**	**0 (0%)**	**28 (19.4%)**
**Multi-organ infection**	**0 (0%)**	**0 (0%)**	**0 (0%)**	**6 (50%)**	**2 (16.7%)**	**7 (58.3%)**	**0 (0%)**	**0 (0%)**	**0 (0%)**	**0 (0%)**	**0 (0%)**	**0 (0%)**	**15 (10.4%)**
**Mouse strain**	**nude** ^**1**^												
**Species**	***P. bovis***			***P. ciferrii***			***P. wickerhamii***			***P. stagnora***			**Total** ^**3**^
**Route^2^**	**I.M.**	**I.P.**	**S.C.**	**I.M.**	**I.P.**	**S.C.**	**I.M.**	**I.P.**	**S.C.**	**I.M.**	**I.P.**	**S.C.**	
Abscess	0 (0%)	5 (41.7%)	5 (41.7%)	0 (0%)	5 (41.7%)	6 (50%)	0 (0%)	0 (0%)	4 (33.3%)	0 (0%)	0 (0%)	0 (0%)	25 (17.4%)
Brain	2 (16.7%)	3 (25%)	0 (0%)	0 (0%)	5 (41.7%)	0 (0%)	1 (8.3%)	2 (16.7%)	2 (16.7%)	0 (0%)	0 (0%)	0 (0%)	15 (10.4%)
Kidney	0 (0%)	1 (8.3%)	2 (16.7%)	0 (0%)	3 (25%)	2 (16.7%)	1 (8.3%)	0 (0%)	0 (0%)	0 (0%)	0 (0%)	0 (0%)	9 (6.3%)
Liver	0 (0%)	2 (16.7%)	1 (8.3%)	0 (0%)	0 (0%)	1 (8.3%)	2 (16.7%)	2 (16.7%)	0 (0%)	0 (0%)	0 (0%)	0 (0%)	8 (5.6%)
Mammary gland	6 (50%)	4 (33.3%)	0 (0%)	5 (41.7%)	2 (16.7%)	2 (16.7%)	8 (66.7%)	5 (41.7%)	0 (0%)	2 (16.7%)	0 (0%)	0 (0%)	34 (23.6%)
Skin	0 (0%)	2 (16.7%)	6 (50%)	0 (0%)	5 (41.7%)	3 (25%)	1 (8.3%)	1 (8.3%)	0 (0%)	0 (0%)	2 (16.7%)	0 (0%)	20 (13.9%)
Spleen	2 (16.7%)	1 (8.3%)	1 (8.3%)	0 (0%)	1 (8.3%)	1 (8.3%)	1 (8.3%)	1 (8.3%)	0 (0%)	0 (0%)	0 (0%)	0 (0%)	8 (5.6%)
**No infection**	**6 (50%)**	**3 (25%)**	**6 (50%)**	**7 (58.3%)**	**3 (25%)**	**5 (41.7%)**	**4 (33.3%)**	**6 (50%)**	**7 (58.3%)**	**10 (83.3%)**	**10 (83.3%)**	**12 (100%)**	**79 (54.9%)**
**Infection (total)**	**6 (50%)**	**9 (75%)**	**6 (50%)**	**5 (41.7%)**	**9 (75%)**	**7 (58.3%)**	**8 (66.7%)**	**6 (50%)**	**5 (41.7%)**	**2 (16.7%)**	**2 (16.7%)**	**0 (0%)**	**65 (45.1%)**
**Single organ infection**	**2 (16.7%)**	**3 (25%)**	**1 (8.3%)**	**5 (41.7%)**	**3 (25%)**	**4 (33.3%)**	**5 (41.7%)**	**3 (25%)**	**4 (33.3%)**	**2 (16.7%)**	**2 (16.7%)**	**0 (0%)**	**34 (23.6%)**
**Multi-organ infection**	**4 (33.3%)**	**6 (50%)**	**5 (41.7%)**	**0 (0%)**	**6 (50%)**	**3 (25%)**	**3 (25%)**	**3 (25%)**	**1 (8.3%)**	**0 (0%)**	**0 (0%)**	**0 (0%)**	**31 (21.5%)**

^1^ In each column, the number of mice which developed *Prototheca* infection in a given organ or overall is provided (each group comprised 12 mice, resulting from pooling of two groups of 6 animals infected with inoculum doses of 10^6^ and 10^7^);

^2^ I.M = intramammary; I.P. = intraperitoneal; S.C. = subcutaneous;

^3^ In each column, the number of mice which developed *Prototheca* infection in a given organ or overall is provided (total number of mice of each strain was 144).

The organs most commonly affected, and at a comparable rate among wt and nude mice, were mammary glands, followed by skin, spleen, kidneys, and liver. The only significant difference between the two mouse strains concerned brain infections, which occurred five times more often in nude mice compared to wt animals (10.4% *vs*. 2.1%; *p* < 0.01; Table 2). Apart from major organs, abscess formation at the injection site was recorded in more than one in ten mice (wt: 11.8%; nude: 17.4%; *p* > 0.05). In wt animals, the abscesses occurred, at a similar frequency, as isolated lesions or combined with other organ lesions (5.6% *vs*. 6.3%), whereas in nude mice the suppurative lesions were more often accompanied by other tissue abnormalities (12.5% *vs*. 4.9%; *p* < 0.05).

Whereas the mouse strain did not significantly affect the frequency of individual lesions, it did influence the extent of their anatomical dissemination. Multi-organ involvement was clearly more common in nude mice compared to wt mice (15/144; 10.4% *vs*. 31/144; 21.5%; *p* < 0.05; Fig 3 and Table 2).

The site of infection was primarily affected by two parameters, namely the algal species and the challenging dose (*p* < 0.001; Table 2). Among the species tested, *P. ciferrii* most frequently caused infections of all tissues analyzed, including kidney (75%), spleen (62.5%), skin (57.9%), abscesses (54.8%), liver (53.3%), brain (38.9%), and mammary glands (38.6%; *p* < 0.001). The rate of infection due to *P. bovis* and *P. wickerhamii* differed across the organs, but never exceeded 30% (Table 2). Finally, *P. stagnora*-induced infections occurred only sporadically and involved either skin (13.2%) or mammary glands (5.3%), with no internal organ involvement (Table 2). Multi-organ involvement was not species-dependent. However, in wt mice, only *P. ciferrii* was able to produce disseminated infection (Fig 3 and Table 2).

In turn, the frequency and dissemination of infection were strongly affected by the infectious dose (*p* < 0.001). The higher dose (10⁷ cells) led to considerably elevated infection rates in both wt (25/144; 17.4% *vs.* 18/144; 12.5%) and nude mice (52/144; 36.1% *vs.* 13/144; 9%) (Fig 3). Likewise, multi-organ involvement increased markedly when the higher dose was used (42/144; 29.2% *vs.* 4/144; 2.8%) (Fig 3).

Irrespective of algal species, challenging dose, or host strain, the IR did not differ significantly in terms of inoculation route. The IRs after I.P., I.M. and S.C. injection of *Prototheca* fell within the ranges of 8.3-58.3%, 9.7-58.3% and 3.4-66.7%, respectively. An exception was *P. stagnora*, which failed to induce infection *via* the S.C. route, while I.M. and I.P. inoculations yielded IRs of 20.8% and 12.5%, accordingly (Fig 3 and Table 2). Although the inoculation route did not significantly affect lesion distribution, infections most often involved tissues adjacent to the injection site, with 74.1% (80/108) of cases confined to this region. Thus, with I.M. and S.C. inoculation, the lesions were predominantly found in the mammary gland (59.6%), and the skin (47.4%), respectively. Whereas purulent lesions invariably developed at the injection site (Table 2). Likewise, the occurrence of multi-organ infections did not differ substantially between the inoculation routes, accounting for 17.7%, 13.5%, and 16.7% of cases following I.P., I.M., and S.C. administration, respectively.

### Post-inoculation clinical monitoring

Throughout the 6-week, post-infection observation period the weight of all animals increased, although the weight gain in nude mice was significantly lower compared to wt animals (+2.91 g *vs*. + 2.32 g; *p* < 0.001). No indications of deterioration in general or behavioral health were observed. However, three of the infected mice, all of the nude phenotype, developed external signs of infection. The lesions (skin erosions or nodules) were found exclusively at the site of inoculation following intraperitoneal or subcutaneous inoculation (Fig 2C).

### Macroscopic changes

Macroscopic changes, as seen upon necropsy, concerned a total of 227 (70.1%) animals, including 87 (53.7%) wt and 140 (86.4%; *p* < 0.001) nude mice. The enlarged lymph nodes and gallbladder were most prevalent in both mouse strains, with those in nude mice being more common (72.3% and 35.8% *vs.* 32.2% and 13%, respectively; *p* < 0.0001). Also, in nude mice, splenic abnormalities were significantly more common than in wt animals (11.7% *vs*. 0.6%; *p* < 0.0001; Fig 2E). Abscesses at the inoculation site also occurred more frequently in nude mice, yet without statistical significance (22.9% *vs*. 15.3%; Fig 2G). Other less common abnormalities (e.g., hyperemia, hypertrophy or steatosis) were observed, at a similar frequency in mice of both phenotypes (*p* < 0.05), in the mammary gland (wt, 6.7% *vs*. nude, 2.5%), liver (2.5% *vs*. 1.9%), and kidney (0.6% *vs*. 0%) (Fig 2F and 2B).

### Microbiology

**Fig 5:**
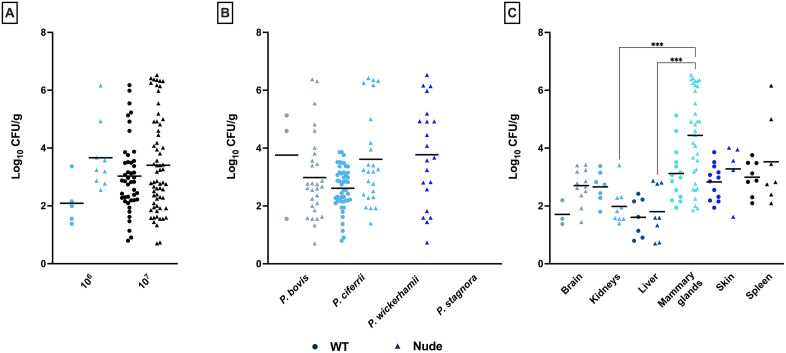
Algal load in tissues (CFU/g) of wt and nude mice categorized by inoculation dose (A), *Prototheca* species used for inoculation (B), and tissue type (C).

The *Prototheca* infection load in the explanted tissues varied among experimental groups according to three parameters, i.e., *Prototheca* species, infection dose, and mouse strain. However, no consistent associations with these variables were found (Fig 5). The organs with the highest algal yield were mammary glands. Here, the mean infection load was calculated at 1.4 × 10⁴ CFU/g and 6.5 × 10⁵ CFU/g in wt and nude mice, respectively. Somewhat lower values were observed for the spleen (wt, 1.9 × 10³ CFU/g; nude, 2.2 × 10⁵ CFU/g) and skin (wt, 1.5 × 10³ CFU/g; nude, 5 × 10³ CFU/g). The brain, kidneys, and liver showed the lowest mean infection loads, ranging from 7.2 × 10 to 7.9 × 10² CFU/g in wt mice, and from 2.6 × 10² to 9.5 × 10² CFU/g in nude mice (*p* > 0.05; Fig 5).

### Histopathology

The frequency and the depth of tissue changes correlated significantly with the algal species (*p* < 0.0001). In general, *P. ciferrii* and *P. bovis* induced more pronounced lesions (Table 3). The highest RISI values were recorded for kidneys (6.8-26.1%), skin (0.5-43.5%), and spleen (5-37.1%). Lower values were noted for the mammary gland (1.6-14.6%), liver (0-9.9%), and brain (0-5%) (Table 3). For most tissues, RISI values did not differ when calculated for tissues positive and negative for *Prototheca.* Exceptions concerned the liver and skin of wt mice, where more intense inflammatory changes were noticed after infection with *P. ciferrii* and *P. stagnora*, respectively (*p* < 0.05). In nude mice, higher RISI values were observed in *P. wickerhamii*-infected mammary glands, and *Prototheca*-free samples of the skin and spleen following *P. bovis* inoculation (*p* < 0.05; Table 3).

**Table 3:** Relative Inflammatory Severity Index (RISI) calculated for six major organs, explanted from mice infected with *Prototheca* species.

	Inflammatory Severity Index^1^																				*p*-value
Mouse strain^2^	**wt**									**nude**										
*Prototheca* sp^3^	***P. b.***		***P. c.***		***P. w.***		***P. s.***		**PBS**	***P. b.***		***P. c.***		***P. w.***		***P. s.***		**PBS**	**Mean**	
Infection status^4^	**+**	**–**	**+**	**–**	**+**	**–**	**+**	**–**	**–**	**+**	**–**	**+**	**–**	**+**	**–**	**+**	**–**	**–**	**+**	**–**
Brain	0	0.03	0.018	0.006	NA	0	NA	0	0.006	0.029	0.002	0	0.002	0.05	0.004	NA	0.035	0.006	0.009	0.008	>0.05
Kidney	NA	0.131	0.117	0.157	NA	0.068	NA	0.243	0	0.194	0.232	0.083	0.136	0.208	0.261	NA	0.195	0.19	0.17	0.128	>0.05
Liver	NA	0.099	0.024	0.016	NA	0.032	NA	0.026	0.028	0	0.014	0	0.018	0.01	0.01	NA	0.01	0.028	0.014	0.029	>0.05
Mammary gland	0.023	0.023	0.146	0.125	0.024	0.035	0.019	0.02	0.016	0.10	0.092	0.10	0.125	0.044	0.039	0.135	0.113	0.021	0.086	0.062	<0.0001
Skin	0.139	0.097	0.014	0.005	NA	0.067	0.074	0.054	0.01	0.368	0.352	0.163	0.137	0.25	0.435	0.375	0.258	0.194	0.159	0.17	<0.0001
Spleen	NA	0.349	0.15	0.124	NA	0.272	NA	0.297	0.207	0.194	0.205	0.05	0.122	0.30	0.262	NA	0.146	0.371	0.167	0.233	<0.05

^1^ NA = not applicable (no infection detected);

^2^ Balb/c wild type (wt) and Balb/c nude mouse strain;

^3^*P. b. = P. bovis*; *P. c. = P. ciferrii*; *P. w. = P. wickerhamii*; *P. s. = P. stagnora*; PBS = control group;

^4^ Infection defined as the presence (+) or absence (-) of *Prototheca* algae in a given organ.

### Comparison of microbiological and histopathological evaluation

In total, *Prototheca* infection was detected in 108 mice. Of these, 49 (45.4%) mice were positive for *Prototheca* infection both on histopathology and microbiological examination. The remaining 59 (54.6%) animals were declared infected on either microbiology (33 or 55.9%) or histopathology (26 or 44.1%) alone. The sensitivities yielded by the two methods were of 75.9% and 69.4%, respectively.

At the tissue level, a total of 160 samples tested positive for infection. Microbiological examination detected algae in 123 tissue samples, while histopathology allowed to prove infection in 85 samples. This translated into sensitivity of 76.9% and 53.1% for microbiology and histopathology, respectively (Fig 3). Overall, the agreement between the two methods was moderate (κ = 0.5; *p* < 0.05), with the concordance level depending on the tissue type, mouse strain, and *Prototheca* species (κ = 0-0.81; *p* < 0.05).

Although microbiological testing generally yielded higher detection rates, infections caused by *P. stagnora* in wt and nude mice and *P. wickerhamii* among wt mice were detected exclusively upon histopathological analysis (Fig 3).

### Blood morphology

Analysis of blood morphology revealed differences associated with mouse strain but not with algal species, infection dose, or route of administration. The levels of WBC, EOS, LYM, and HGB were significantly higher in wt mice compared to nude mice (*p* < 0.05; Table 4). In turn, MCH and MCV were higher in the latter animals (*p* < 0.0001). Apart from a slight reduction in MCV in wt mice (*p* < 0.05), *Prototheca* infection did not affect hematological parameters, including WBC, LYM, NEU, MONO, EOS, and BAS. Noteworthy, a consistent decrease in HGB and leukocyte counts (including WBC, LYM, EOS, and BAS) was observed in all experimental animals when compared to the reference ranges provided by the breeding facility (Table 4).

**Table 4:** Blood morphology of *Prototheca*-infected mice.

	Infection						
	Negative			Positive			Ref.^2^
Mouse strain^1^	wt	nude	*p*-value	wt	nude	*p*-value	
Red Blood Cells [T/L]	9.99	9.54	<0.0001	10.01	9.53	<0.01	10.5 ± 0.4
Hemoglobin [g/dL]	15.18	14.97	>0.05	15.24	15.86	<0.05	16.4 ± 0.6
Mean Corpuscular Volume [fL]	46.41	48.39	<0.0001	46.45	48.41	<0.0001	55 ± 1
Mean Corpuscular Hemoglobin [pg]	15.30	15.56	<0.0001	15.33	15.56	<0.0001	15.8 ± 0.4
Blood Plates [g/L]	1303.13	1238.89	>0.05	1296.50	1310.98	>0.05	1137 ± 172
White Blood Cells [g/L]	4.88	3.56	<0.0001	4.86	3.09	<0.0001	11.0 ± 2.8
Neutrophils [g/L]	0.92	1.01	>0.05	0.91	0.91	>0.05	1.21 ± 0.23
Lymphocytes [g/L]	3.7	2.31	<0.0001	3.32	2.08	<0.001	8.67 ± 2.28
Eosinophils [g/L]	0.13	0.07	<0.0001	0.12	0.06	<0.0001	0.45 ± 0.14
Monocytes [g/L]	0.13	0.15	0.05	0.12	0.13	>0.05	0.16 ± 0.08

^1^ Balb/c wild type (wt) and Balb/c nude mouse strain;

^2^ Reference values provided by the breeder for Balb/cAnNRj mouse strain (Janvier Labs).

### Cytokine profiling

**Fig 6:**
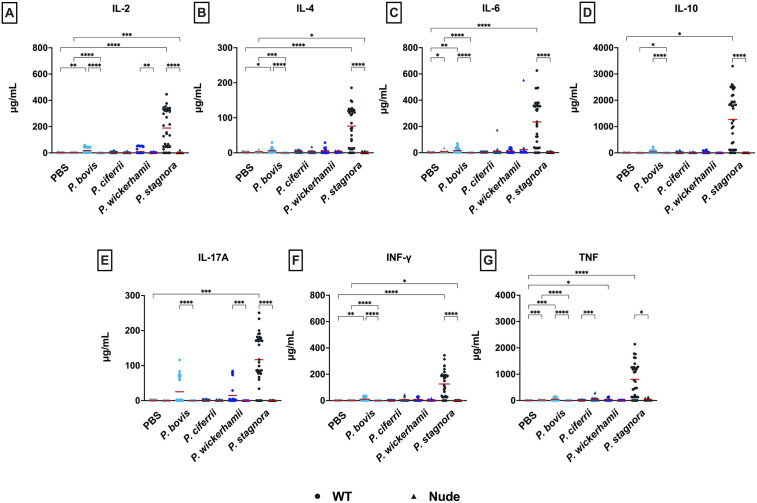
Levels of seven different cytokines (A-G) measured in blood samples collected from wt and nude mice at euthanasia, categorized by the *Prototheca* species used for infection.

The cytokine profile differed markedly between mouse strains, with wt animals consistently exhibiting higher concentrations of both proinflammatory and regulatory cytokines (*p* < 0.05; Fig 6), regardless of other infection parameters (algal species, inoculum dose, route of administration). The most pronounced differences were observed for IL-10 (334 pg/mL *vs*.1.6 pg/mL), TNF (221 *vs*. 26 pg/mL), and IL-6 (67 *vs*. 10 pg/mL). Other cytokines, including IL-2, IL-17A, IFN-γ, and IL-4, were likewise elevated in wt mice, although to a lesser extent (22.3-55 pg/mL *vs*. 0.2-2.8 pg/mL).

Of the four *Prototheca* species tested, the most marked cytokine response was elicited by *P. stagnora*, albeit only in wt mice (80-846 pg/mL; Fig 6). In contrast, cytokine levels in nude mice infected with this algal species remained comparable to controls (0.2-31.4 pg/mL *vs.* 0-18.8 pg/mL). The remaining *Prototheca* species elicited overall weak cytokine responses, with their levels in wt mice not exceeding 50 pg/mL (3.1-48.1 pg/mL) and comparably low concentrations observed in nude mice (0-58.8 pg/mL; Fig 6). No evident differences in cytokine levels were observed in any of the experimental groups, as far as the route of inoculation, infectious dose, or disease progression were concerned.

## Discussion

Studying the pathogenicity of *Prototheca* algae *in vivo* is very challenging. This is due to the lack of a reliable and validated animal model and the still rudimentary understanding of *Prototheca* biology. A limited number of previous investigations have produced variable and inconclusive results [17–40]. The present study is the first attempt to develop a comprehensive and robust animal model of protothecosis, whose experimental path was established using a multifactorial design, with a multi-cohort study sample. For the first time, the effects of different algal species, inoculation dose, and route of inoculation, as well as the immunological background of the host, on the development and severity of *Prototheca* infection in a murine model were systematically explored.

In this study, all *Prototheca* species examined were able to produce infection in mice, yet the infection potential differed between the species, depending on the experimental conditions. The overall infection rate was calculated at 37.5%, with the highest rate for *P. ciferrii* (61.1%), followed by *P. bovis* (45.8%), and *P. wickerhamii* (31.9%). Whereas, *P. stagnora* caused infections merely in single animals (11.1%). The pathogenic capacity of *Prototheca* species was reflected not only in the frequency of infections but also in the range of tissues affected. *P. ciferrii* produced infections in all organs examined, demonstrating broad tissue tropism. *P. bovis* was likewise recovered from various organs, albeit the frequency and extent of the lesions were somewhat lower than for *P. ciferrii*. Contrastingly, infections caused by *P. wickerhamii* were generally mild and mostly confined to the mammary gland tissue. The few infections due to *P. stagnora* were of limited severity and were typically localized at peripheral sites, such as the skin or mammary glands.

All four *Prototheca* species evaluated in this study had previously been shown to exhibit pathogenic potential, although these earlier findings have originated from only a handful of anecdotal studies (Table 1). For *P. stagnora* in particular, the evidence comes from a single study, which involved intratesticular inoculation of various host species [22]. Only four *in vivo* studies have directly assessed the pathogenic capacity of different *Prototheca* species [20,22,30,40], yet one study alone has identified a clear interspecies distinction, with *P. zopfii* (currently recognized as *P. bovis* or *P. ciferrii*) causing about 1.7 times more infections than *P. wickerhamii* [30].

Differences in virulence between *Prototheca* species may relate to variation in metabolic plasticity and mechanisms of intracellular persistence. A comparative genomic and transcriptomic study indicated that highly pathogenic *P. bovis* strains show enhanced expression of genes involved in adaptation to phagolysosomal stress and survival within macrophages [41]. Likewise, previous studies on *P. wickerhamii* have identified substantial strain-level differences in gene expression [42,43], including genes involved in cell wall biosynthesis and remodeling, such as those encoding mannan endo-1,4-β-mannosidase. Downregulation of this enzyme resulted in thinning of the cell wall and reduced cytotoxicity toward macrophages [43]. This, in turn, may contribute to altered interactions with host immune cells, potentially affecting susceptibility to immune clearance. Such variability in virulence-associated characteristics may also underlie the differences in infection severity observed between species in our study. However, further comparative multiomic studies are necessary to provide a definitive explanation for interspecies variation in virulence.

In this work, a higher inoculum dose (10^7^) was associated with an increased infection rate. This correlation was particularly evident for *P. ciferrii* in both mouse phenotypes. *P. bovis* and *P. wickerhamii* followed the same dose-dependent pattern, but only in nude mice. In contrast, infection rates with *P. stagnora* remained low regardless of the inoculum size (Fig 3). The trend, linking a larger inoculum with a higher infection rate, had previously been reported, albeit in only one study, where intravenous inoculation of mice with *P. zopfii* resulted in fewer infections when the inoculum was reduced from 10^5^-10^7^ to 10^3^-10^4^ [27].

As for the inoculation route, I.M. and I.P. applications were markedly more effective than S.C. injection. This was observed across all tested *Prototheca* species and mouse strains (Fig 3 and Table 2). Similar to our findings, I.M. inoculation proved to be the most effective route for infecting mice in previous investigations [17,18,32,34] Meanwhile, the I.P. route resulted in infection in over half of the challenged animals, as demonstrated across earlier investigations [20–22,25,30,32,36,39].

In the present study, the infections remained largely subclinical, and only a small subset (2%) of nude mice developed visible lesions, such as skin erosions or nodules at the injection site, after I.P. or S.C. administration. No mortality was reported, and the overall clinical condition of both wt and athymic mice remained stable throughout the whole observation period. Single-organ infections occurred at relatively similar rates in both wt and nude mice, hovering around 20% in both groups. Multi-focal infections were slightly less common, affecting every tenth wt mouse and every fifth nude mouse (Table 2). The frequency of such systemic infections was 10-fold greater when the higher inoculation dose was applied. Neither the inoculation route nor the *Prototheca* species affected the dissemination pattern of infection.

As shown in earlier research, clinical signs were generally absent, although one study noted a dose-dependent increase in localized redness and swelling at the injection site [32]. Lethal outcomes were reported in at least twelve animals across four investigations irrespective of whether or not the disease had disseminated [19,21,27,40]. Only a single study attributed mortality to I.V. administration and a high inoculum dose (10⁷) [27]. A few studies documented disseminated *Prototheca* infections in an animal model. These infections concerned rabbits, cows, and mice, exposed to inocula of 10^3^-10^7^ *P. ciferrii*, *P. wickerhamii* or *P. zopfii* given via I.P., I.T. or I.V injection [19,25,27,30,36].

Algal loads in our experiments averaged from 7.2 to 6.5 × 10^5^ CFU/g in different organs, with the highest mean count observed for the mammary glands and the lowest for the liver. In contrast, former animal studies reported considerably higher tissue burdens, ranging from 1 × 10⁴ to 5 × 10⁵ CFU/g of infected tissue [17,18,32,34]. Interestingly, some studies comparing tissue colonization in mice sacrificed at different post-infection times showed a gradual reduction in algal load over time, a trend consistently observed across different *Prototheca* species and under various experimental conditions [22,32].

As with other opportunistic pathogens, vulnerability to *Prototheca* infection and severity of the disease has usually been considered as a function of the immunological status of the host. Among the factors predisposing the development of protothecosis, corticosteroid-induced immunosuppression, neutrophil dysfunction, and impaired T-cell based immunity have most frequently been speculated [23,44,45]. On the other hand, the relatively low prevalence of the disease in HIV-positive and AIDS patients has cast doubt over the role of T cells as critical drivers of the pathogenesis of protothecosis [8,46]. The few animal-based studies evaluating the immune competence of the host have yielded ambiguous and inconclusive results, as some showed higher infection rates in immunocompromised animals versus healthy ones, while the others found the reverse [23,25,27,33].

Although there is no consensus on whether *Prototheca* algae are intracellular or extracellular pathogens, the literature provides several lines of evidence that the *Prototheca* lifestyle in the host involves intracellular residence and proliferation. *Prototheca* have been demonstrated to survive and replicate within phagocytic compartments of macrophages and neutrophils [44,47,48]. The algae have also been shown to invade and persist inside epithelial cells [17,49]. Furthermore, the gene expression profile for pathogenic *Prototheca* strains differed from that of environmental strains, reflecting a response adapted to the intracellular milieu [41]. From these observations, it was conjectured that a T cell-mediated response might be primarily involved in *Prototheca* infection. Consistent with this hypothesis, athymic mice, deficient in T lymphocytes, were employed in this study.

Compared to wt mice, the immunosuppressed animals were 1.5 times more susceptible to *Prototheca* infection with an overall infection rate of 45.1%. Likewise, disseminated infections in nude mice occurred twice as often as in the control animals. These observations appear to argue in favor of the importance of T cell-mediated immunity. Still, half of the athymic mice did not develop infection, which may allude to the role of innate immune response, including phagocytosis by neutrophils and macrophages, and the complement pathway, particularly in the early stages of infection. In this context, it is worth noting that *in vitro* studies on *Prototheca* infection have yielded somewhat conflicting results. While some have demonstrated that human and murine phagocytes can internalize *Prototheca* cells, with this process being species-dependent and often enhanced by IgG-mediated opsonization [17,48,50], the others have indicated that bovine and murine phagocytes may fail to effectively control algal proliferation [44,51]. The scenario is further complicated by the fact that the susceptibility to *Prototheca* infection and the defective response to *Prototheca* antigens in nude mice, as a result of lymphocytic depletion, can be mitigated by the activity of still-functional B cells and a relatively normal IgM response to thymus-independent antigens. Thus, to better assess the capacities of isolated cell populations for rendering protection against *Prototheca* infection, studies employing severe combined immunodeficiency (SCID) mice, lacking both T and B lymphocytes as models, should be considered. Whereas, mouse strains with neutrophil or natural killer (NK) cell deficiencies may provide useful models for clarifying the role of non-specific immunity components [52–54].

Previous studies, both *in vitro* and *in vivo*, have demonstrated elevated cytokine levels following *Prototheca* infection, typically peaking within 12-24 hours post exposure [17,18,34]. The cytokines whose secretion increased consistently in response to *Prototheca* infection included TNF-α and IL-10 [17,18,34]. These were also found elevated in the present study, with higher values in wt than athymic mice. This difference may indicate a substantial contribution of T lymphocytes in wt mice to cytokine regulation, since both TNF-α and IL-10 can be secreted by activated T cells in addition to macrophages. Sustained TNF-α expression may contribute to infection control by promoting macrophage activation and inflammatory signaling, whereas IL-10 counteracts these effects by limiting macrophage activation and antigen presentation [55]. Interestingly, IL-10 is also a hallmark product of regulatory T cells, which maintain immune homeostasis through ongoing activity in response to commensal microbiota and endogenous antigens [56,57]. Thus, the elevated IL-10 observed in response to *P. stagnora* may reflect a regulatory rather than pro-inflammatory immune function, consistent with the normally saprophytic (non-pathogenic) phenotype of this species.

Other immune mediators, including IL‑2, IL‑4, IL‑6, and IL‑17A, showed only minor increases. Captivatingly, wt mice infected with the saprophytic *P. stagnora* showed conspicuously higher cytokine responses than those challenged with pathogenic *Prototheca* species, suggesting that other factors are involved in shaping the response to the pathogen and channeling the cascade of the inflammatory events and other pathological processes at the cellular level. Such factors, which may differ between species, could include, cell-surface-associated antigens. This speculation is supported by findings in *Candida albicans*, where cell wall remodeling has been shown to attenuate immune detection and cytokine induction [58,59].

Finally, a comment should be made in regard to the identification of protothecosis in an animal model. Canonically, the diagnostic path for *Prototheca* spp. includes histopathological examination and tissue culture, both of which were evaluated in this study. Less than half of the mice that had developed *Prototheca* infection were positive upon histopathology and microbiology, while the remaining infections were detected by either method. Although culture achieved about 10% higher sensitivity, histopathology was the only examination that enabled identification of *P. stagnora*. These findings align with earlier reports, which highlighted technical challenges in *Prototheca* diagnostics, particularly when the algal load is low or cell viability is compromised [8,22,60–62]. Accordingly, the combined use of microbiological and histopathological methods improves diagnostic reliability.

## Conclusions

To conclude, this study provides new insights into experimentally induced protothecosis in mice, emphasizing how the pathogen species, the inoculation dose and route, and the host immune status all impact the development of infection. The findings from this investigation can be distilled into four key messages. First, pathogenic capacity differs between species, with *P. ciferrii* being the most virulent and *P. stagnora* showing the lowest virulence. Second, host immune competence affects the development and spread of *Prototheca* infection, with athymic mice being more susceptible to infection and to multifocal involvement over wt mice. Third, higher inocula (10⁷) were associated with higher infection yield, whereas the route of administration influenced the site of infection but not its severity. Fourth, the combined use of microbiological and histopathological methods enhanced the detection of *Prototheca* algae in host tissues. Overall, this study underscores three features of the protothecal disease, namely species-specific pattern of pathogenicity, chronic, asymptomatic or asymptomatic infection as a predominant clinical manifestation, and a pivotal role of the host immunological background in shaping the course and severity of the disease. The findings from this work provide a template for future animal model studies, advancing our understanding of *Prototheca* pathogenicity and guiding strategies to investigate the virulence mechanisms of this peculiar pathogen. Future research is expected to elucidate the mechanisms of *Prototheca* virulence and host-pathogen interactions. The use of different immunodeficient models is thought to provide a better understanding of the roles of adaptive and innate immunity in protothecosis.

## Supporting information

S1 Table Scalar assessment of the intensity of the identified histopathological changes – International Harmonization of Nomenclature and Diagnostic (INHAND) criteria. (XLSX)
